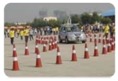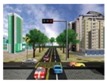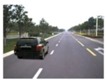# Simulating Dynamic Driving Behavior in Simulation Test for Unmanned Vehicles via Multi-Sensor Data

**DOI:** 10.3390/s19071670

**Published:** 2019-04-08

**Authors:** Danchen Zhao, Yaochen Li, Yuehu Liu

**Affiliations:** 1 Institute of Artificial Intelligence and Robotics, Xi’an Jiaotong University, No. 28 Xianning West Road, Xi’an 710049, Shaanxi, China; danchenzhao@foxmail.com; 2 School of Software Engineering, Xi’an Jiaotong University, No. 28 Xianning West Road, Xi’an 710049, Shaanxi, China; yaochenli@mail.xjtu.edu.cn

**Keywords:** simulation test, dynamic driving behavior, traffic scene augmentation, corridor model

## Abstract

Driving behavior is the main basis for evaluating the performance of an unmanned vehicle. In simulation tests of unmanned vehicles, in order for simulation results to be approximated to the actual results as much as possible, model of driving behaviors must be able to exhibit actual motion of unmanned vehicles. We propose an automatic approach of simulating dynamic driving behaviors of vehicles in traffic scene represented by image sequences. The spatial topological attributes and appearance attributes of virtual vehicles are computed separately according to the constraint of geometric consistency of sparse 3D space organized by image sequence. To achieve this goal, we need to solve three main problems: Registration of vehicle in a 3D space of road environment, vehicle’s image observed from corresponding viewpoint in the road scene, and consistency of the vehicle and the road environment. After the proposed method was embedded in a scene browser, a typical traffic scene including the intersections was chosen for a virtual vehicle to execute the driving tasks of lane change, overtaking, slowing down and stop, right turn, and U-turn. The experimental results show that different driving behaviors of vehicles in typical traffic scene can be exhibited smoothly and realistically. Our method can also be used for generating simulation data of traffic scenes that are difficult to collect.

## 1. Introduction

Evaluation of the intelligence level and comprehensive performance of unmanned vehicles turns to ontology and phenomenology. According to Turing [1], a system could be said to be intelligent enough for special kind of tasks if, and only if, it could finish all the possible tasks of its kind. Therefore, we can begin to achieve safe and reliable artificial intelligence (AI) systems if, and only if, the tests have clear definitions of tasks and efficient methods to generate abundant data for tests. As a result, appropriate AI testing methods should be task-driven and data-centric [2].

Driving behavior is the main basis for evaluating the performance of unmanned vehicles. Testing the driving behaviors of unmanned vehicles is an important means of giving scientific and just evaluation of the research quality of key technologies such as environment perception, control, and decision [3]. Unmanned vehicles should be tested in a typical traffic environment including static, dynamic, and uncertain factors such as urban roads, highway, and rural roads.

The most authoritative way to test and verify unmanned vehicles is by testing on the real road. For this purpose, competitions are held all over the world, such as DARPA-Urban Challenge in the US and the Future Challenge in China. However, this kind of test method is faced with difficulties such as limitation of test site, unrepeatable test condition, time-consuming, and high-cost procedure. Additionally, the actual testing environment may not be accessible, or may only be accessible at certain times while the simulated environment is always available [4]. Therefore, there is a growing trend in unmanned vehicle research to use a simulated environment and several simulation and test platform (STP) are established. But most of them use virtual data instead of real data collected from actual world which will reduce the reliability of the simulation result. The comparison of three test methods based on different data types is shown in Table 1.

**Figure 1:**
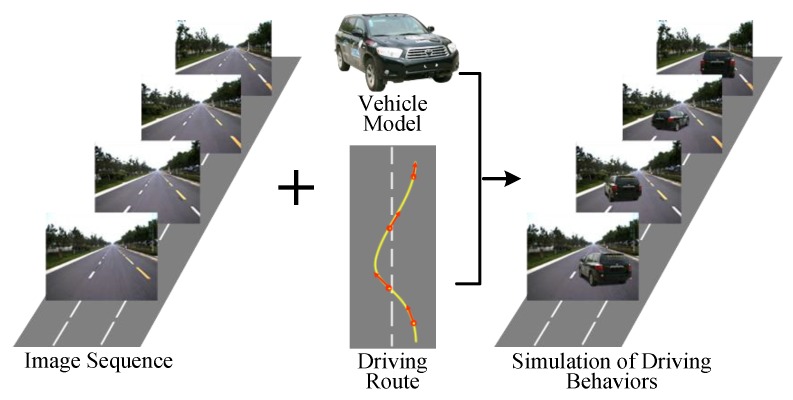
Simulation of driving behaviors with image sequences collected from real road environment.

Faced with these limitations, we try to explore ways to realistically simulate and exhibit typical driving behaviors of vehicles with real data of traffic environment in simulation test of unmanned vehicles, as shown in Figure 1. We use the real multi-sensor data captured from the real traffic environment and augment the traffic scene with vehicles in different driving behaviors.

However, there are quite a few challenges for us to achieve this goal. One is the modeling of virtual vehicles. For “traditional” computer graphics, recent advances in material modeling and global illumination have facilitated the synthesis of realistic and detailed imagery. But it needs painstaking work for the actual visual world, which is very complicated. Each object to be rendered requires lots of work to give surface properties and detailed geometry. Though we can use many image-based approaches to build the model, the data is not acquired straightforward and the methods are not suitable for our relatively large and moving vehicles. Another one is the spatial topology relationship between the virtual vehicles and the road environment. The scene for testing the unmanned vehicles is an image sequence containing spatial topology information. Virtual vehicles have spatial topology attributes, i.e., virtual vehicles should be consistent with reference scene in both appearance and spatial topology relationship.

Encouragingly, we have proposed a simple and effective approach which can exhibit typical dynamic driving behaviors smoothly and realistically.

## 2. Overview

As part of our work on parallel testing of vehicle intelligence [2], we propose an automatic approach of simulating dynamic driving behaviors of vehicles. The spatial topological attributes and appearance attributes of virtual vehicles are computed separately according to the constraint of geometric consistency of sparse 3D space organized by image sequences.

There are three critical elements for us to solve our task:

*Typical Traffic Scene*: Unmanned vehicles should be tested in a typical traffic environment including static, dynamic, and uncertain factors such as urban roads, highways, and rural roads. We analyze test tasks and scoring criteria of DARPA-Urban Challenge and the Future Challenge. Then, a typical traffic scene including the intersections is chosen for virtual vehicles to execute the driving tasks of lane change, overtaking, slowing down and stop, right turn, and U-Turn.

**Figure 2:**
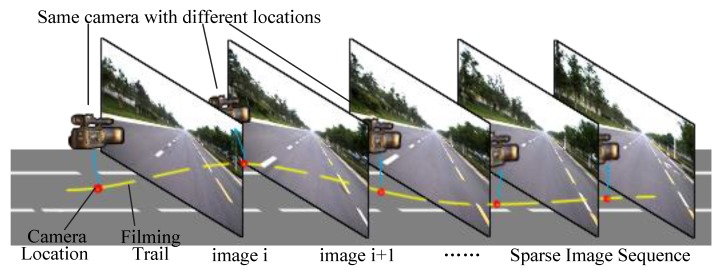
Representation of reference road scene using sparse ordered image sequence containing data of real filming location of viewpoints.

*3D Road Environment Representation*: Road scene simulation and modeling based on vehicle sensors are currently an important research topic in the field of intelligent transportation systems [5,6,7,8]. Most current image compositing approaches treat 3D road environment representation as a 2D problem, such as Photoshop. As described by Lalonde et al. [9], we agree with their point of view that any image manipulation must be done in the 3D space of the scene, not in the 2D space of the image. But it is a pity that Lalonde et al. did not precisely define and describe that 3D space of scene. We believe that the three-dimensional road environment is a data field containing image data, spatial topology data, and motion data. Generated novel scene video visualizes these data. As shown in Figure 2, the reference road scene is represented by a sparse ordered image sequence containing data of real filming location of viewpoints. Recent advances in camera calibration, 3D registration, and scene reconstruction have allowed the synthesis of not only images and videos, but also the data of spatial topology. In this paper, we use the GPS coordinates of both viewpoint and virtual vehicles to compute the transformation parameters and open-source software OSM2World to transform a map description exported from OpenStreetMap into a mesh model of road surface. To restrict the motion range of virtual vehicle to the road area and simplify coordinate transformation, we defined a “corridor model” and applied triangle collision detection based on Irrlicht engine [10,11,12].

*Geometric consistency of vehicle model and road environment*: Humans can easily recognize a synthesized object when observing an image. What are the criteria for us? One can judge whether the object should be placed at this location, whether the object should be looked like a side view or a front view, and whether the size of object is fit, too big, or too small. Then, it is easy to understand that the main manifestation of the geometric consistency is that the vehicle should have appropriate position, pose, and size in visualization. In order to solve this problem, we firstly specify the model for virtual vehicle and road environment. The road environment is organized by image sequences. For each image, extrinsic and intrinsic parameters of camera at the corresponding viewpoint are known. For the virtual vehicle, we combine 3D model with multi-viewpoints corresponding to different vehicle images.

## 3. Related Works

Typical traffic scene: Work of designing and building typical traffic scene for unmanned vehicle tests are being done by researchers. Based road traffic accident data from the years 2000–2010 and from several aspects such as human, vehicle, road environment, and accident form, Zhou et al. [13] selected the greatest impacts of traffic safety to make up the typical dynamic traffic event. In their research, one type of dynamic events in city road scene was selected as a case, which is the conflict between the main vehicle and pedestrians in front of parking bus when pedestrians crossing street on the main road in city. In June, researchers at the University of Michigan announced that they are in the process doing building a fake city center. According to a press release, the fake city center locates on a 13-hectare plot at the school’s North Campus just outside Ann Arbor. The faux downtown, to be known as the mobility transformation facility (MTF), will have a four-lane highway, stoplights, intersections, roundabouts, road signs, a railroad crossing, and construction barrels. The facility’s designers are also putting up building facades meant to simulate the challenge of transmitting wireless signals inside urban canyons [14].

Prior scene maker: A survey about internet visual media processing [15] showed a number of recent papers demonstrated the work on visual content maker. Lalonde et al. [9] presented the photo clip art system for inserting objects into an image. Users can insert object by specifying a class and a position for the inserted object, which is selected from a clip art database by matching various criteria including camera orientation and lighting conditions. However, their research works on static images rather than continuous dynamic scenario video. Besides, only specific images can be synthesized. For certain objects, not all the possible appearances are available. Eitz et al. [16] proposed a PhotoSketcher system with the goal of synthesizing a new image only given user drawn sketches. The synthesized image is blended from several images, each found using a sketch. However, users must put additionally scribbles on each retrieved image to segment the desired object. The above methods are limited to the synthesis of a single image. To achieve synthesis for image sequence (e.g., scene video as mentioned before), the main additional challenge is to maintain consistency of the same vehicle across successive frames, since candidates for all frames usually cannot be found in the database. Chen et al. [17] proposed the PoseShop system, intended for synthesis of personalized images and multi-frame comic strips. It first builds a large human pose database by collecting and automatically segmenting online human images. Personalized images or comic strips are produced by inputting a 3D human skeleton. Head and clothes swapping techniques are used to overcome the challenges of consistency. However, the PoseShop system did not work very well on dealing with the accurate spatial topology relationship between the background scene and synthesized objects so that geometric consistency criteria can’t be matched. Flagg et al. [18] presented a system for capture, analysis, synthesis, and control of video-based crowds. They introduced crowd tubes samples and constraint violations with a conflict graph to avoid collisions. Abdi et al. [19] augmented the traffic signs to provide visual feedbacks to drivers for an enhanced driving experience. They used a virtual 3D model, with a known size, to define a reference coordinate system. They projected the 3D object sign using the corresponding sparse dictionary. Their augmentation is for enhancing driving experience and lacks of reality. The above researches suggest that efforts in this direction are very timely.

The rest of the paper is organized as follows: The geometric consistency of 3D vehicle model and road environment is presented in Section 4. In Section 5, the construction of road scene corridor model and traffic scene augmentation is introduced. Experiments and comparisons are shown in Section 6. Finally, we close this paper with conclusion and future works.

## 4. Geometric Consistency of 3D Vehicle Model and Road Environment

Vehicle images that match geometric consistency criteria can be obtained by three steps. First, the virtual vehicle should be registered to the 3D road space. Second, the vehicle pose (i.e., vehicle image) corresponding to different viewpoints is obtained. Finally, we compute the scale factor to give the vehicle image an appropriate size.

### 4.1. Registering 3D Vehicle Model to Road Scene

The position of virtual vehicle and image sequence are not always coincident. So, first of all, we should register the virtual vehicle to a proper position. That is to say, while the real vehicles are at varying distances from the user, the virtual vehicles are all projected to the same distance.

Techniques of augmented reality [20] contribute to this task. By techniques of 3D registration, we can obtain the position of virtual vehicle in the image of road scene. First, we should compute the location of virtual vehicle in road scene. That is, to compute the virtual vehicle’s coordinates in virtual 3D space formed by image sequences.

In the real road environment, the road plane is denoted by *x*-*y* plane. Each captured image combines with a viewpoint’s coordinate *P*_1_ = (*x*_1_, *y*_1_, *z*_1_). The coordinate of the virtual vehicle’s center is *P*_2_ = (*x*_2_, *y*_2_, *z*_2_). According to the assumption of *x*-*y* plane, *z*_1_ is the vertical height of viewpoint to the road plane while *z*_2_ is the vertical height of virtual vehicle’s center to the road plane. *P_v_* = (*x*, *y*, *z*) is the coordinate of virtual vehicle’s center in the coordinates of virtual 3D road space. Since the *z* axes of three coordinates are in parallel with each other, we can get that *z*=*z*_2_-*z*_1_. Then, we will compute the *x* and *y* coordinates of *P_v_*.

**Figure 3:**
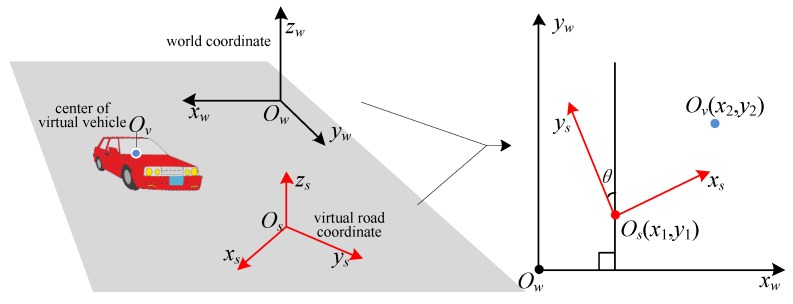
Coordinate transformation between the coordinates of real road environment and the coordinates of virtual road scene.

We assume that the coordinate origins of real road environment, virtual road scene, and vehicle model are *O_w_*, *O_s_*, and *O_v_* respectively. As shown in Figure 3, the angle between *y_s_* and *y_w_* is *θ*.

If we make $P_{1}^{C}={\left[x_{1},y_{1},1\right]}^{T}$, $P_{2}^{C}={\left[x_{2},y_{2},1\right]}^{T}$ and $P^{C}={\left[x,y,1\right]}^{T}$, then we can get

(1) $$P^{C}={}_{A}^{B}{RP}_{2}^{C}+P_{1}^{C}$$

where ${}_{A}^{B}R$ is the rotation matrix for *w* coordinates to the *s* coordinates, and

(2) $${}_{A}^{B}R=\left(\begin{array}{ccc} \cos\theta & \sin\theta & 0\\ -\sin\theta & \cos\theta & 0\\ 0 & 0 & 1 \end{array}\right).$$

After the 3D coordinate of virtual vehicle’s center is obtained, we can use it to get the center’s coordinate in each image. If the coordinate of virtual vehicle’s center in the image is (*u*, *v*), and the projection matrix for 3D to 2D coordinates is ***P***, then we have

(3) $$\left[\begin{array}{c} u\\ v\\ 1 \end{array}\right]=P_{3\times 4}\left[\begin{array}{c} x\\ y\\ z\\ 1 \end{array}\right]$$

where $P_{3\times 4}=A_{3\times 3}T_{3\times 4}$. $A_{3\times 3}$ is the intrinsic parameters while $T_{3\times 4}$ is the extrinsic parameters of the camera. The intrinsic and extrinsic parameters of the camera can be obtained by trilinear method [21].

### 4.2. Vehicle Image in Road Scene

Obtaining the image of virtual vehicle in the road scene consists of two parts, the view image and the scale transformation. The view image shows the virtual vehicle’s pose in real time when the vehicle is moving in the road scene. Through the scale transformation, the view image can be synthesized with appropriate size.

#### 4.2.1. The View Image of 3D Vehicle Model

**Figure 4:**
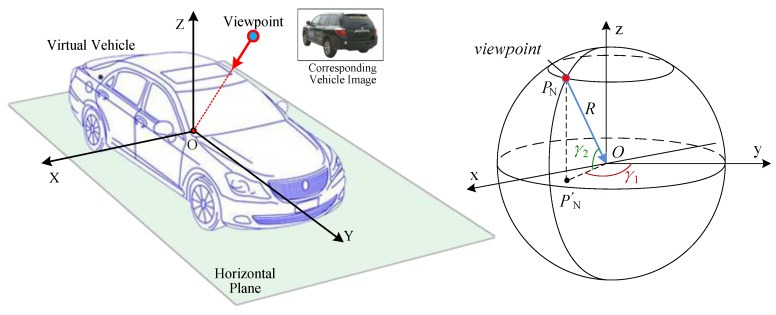
3D vehicle model combined with multi-viewpoints.

In the 3D vehicle model combined with multi-viewpoints, the distance between the viewpoint and the center of virtual vehicle varies with different viewpoints. For ease of management, we normalize all the viewpoints onto a spherical surface. The center of sphere coincides with the center of virtual vehicle. The spherical radius is *R*. Figure 4 shows the normalized viewpoint sphere. Each viewpoint lies on the sphere equidistant from the center is denoted by two variables, that is *P_N_* = (*γ*_1_, *γ*_2_).

When *γ*_1_ and *γ*_2_ are known, the view image corresponding to the viewpoint can be retrieved. That is to say, all the view images of virtual vehicle can be indexed by two variables. As described above, the coordinates of viewpoint and virtual vehicle are *P*_1_ = (*x*_1_, *y*_1_, *z*_1_) and *P*_2_ = (*x*_2_, *y*_2_, *z*_2_) in real road environment respectively. If the unit vector of the *y* axis of vehicle model in real traffic environment is (*v_i_*, *v_j_*), we can obtain

(4) $$\gamma_{1}=\arctan\frac{y_{1}-y_{2}}{x_{2}-x_{1}}-\arctan\frac{v_{i}}{v_{j}}$$

(5) $$\gamma_{2}=\arctan\frac{z_{1}-z_{2}}{\sqrt{{\left(x_{1}-x_{2}\right)}^{2}+{\left(y_{1}-y_{2}\right)}^{2}}}.$$

#### 4.2.2. Scale Transformation

**Figure 5:**
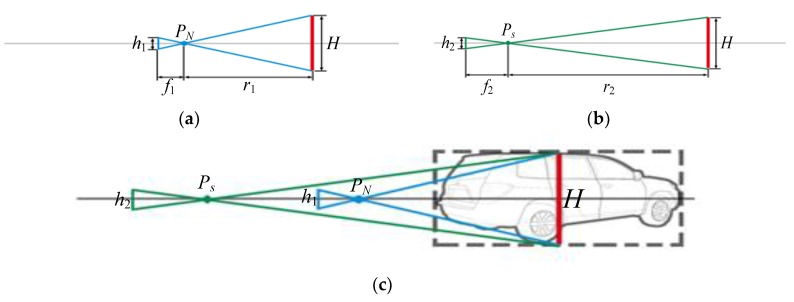
View space of vehicle model computation with scale factor using pin-hole model. (**a**) Pin-hole model at the viewpoint of *P_N_* which lies on normalized spherical surface; (**b**) pin-hole model at the viewpoint of *Ps* which is in the 3D space of road scene; (**c**) overlapping the optical axis in (a) and (b), the scaling relation is clear.

After the view image of virtual vehicle is retrieved, we transform its scale to make it fit the scene with appropriate size. The normalized views have the same scale. Based on the model in Figure 4, if *P_N_* is definite, the sight line of viewpoint *P_s_* in virtual road space coincides with that of the viewpoint lies on normalized spherical surface (i.e., *OP_N_*). Based on principle of pin-hole imaging, the sight lines are coincided as that is shown in Figure 5.

For the viewpoint lying on normalized spherical surface (i.e., *P_N_*), the focal length of camera is *f*_1_, and the size of vehicle image is *h*_1_. The distance from *P_N_* to the center of vehicle is *r*_1_. For the viewpoint (i.e., *P_s_*) in virtual road space, the focal length of camera is *f*_2_, and the size of vehicle image is *h*_2_. The distance from *P_s_* to the center of vehicle is *r*_2_. Then we can easily get

(6) $$h_{2}=\frac{r_{1}f_{2}}{r_{2}f_{1}}h_{1},$$

where $r=\sqrt{x^{2}+y^{2}+z^{2}}$.

So the scale factor is *r*_1_ *f*_2_/(*r*_2_ *f*_1_).

## 5. Visual Simulation of Driving Behaviors

In the traffic scene, both static traffic elements that can influence scene semantics such as traffic signs and the subject of traffic flow (vehicle) need explicit geometric description of the road surface to support their structures and motion. On the basis of obtained trail of viewpoint locations corresponding to image sequences, GIS data of the road can be obtained by GIS (geographic information system) or open-source map such as OpenStreetMap [22]. In GIS data, different layers are used to represent the geographical characteristics. A road is represented by a polyline formed by a set of points of geographical locations. In addition, road attributes can be described by parameters such as name, road type, width, and number of lanes.

**Figure 6:**
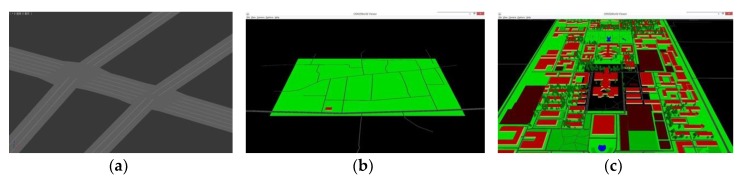
Three kinds of road model built from geographic information system (GIS) data. (**a**) Roads with details such as lanes and intersections; (**b**) roads in a region (data captured from suburb of Xi’an, China); (**c**) model with information besides of road (data captured from Xi’an Jiaotong University, Xi’an, China).

In practical work, in order to improve efficiency of modeling, we use open-source software OSM2World to transform map description exported from OpenStreetMap into mesh model of road surface, shown in Figure 6.

**Figure 7:**
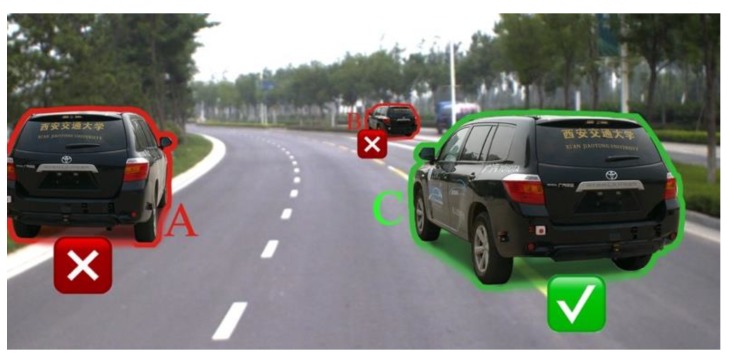
Augmented traffic scene. Vehicle A and B appear at illogical location (tree lawn).

The method in Section 4 and Reference [7] is a pervasive vehicle synthesis method for augmented traffic scene. However, without constraint of road space structure, the synthetic vehicle may appear at illogical location in the road scene, shown in Figure 7. Thus, we propose a logical model named as “corridor model” to restrict the motion range of virtual vehicles.

### 5.1. Corridor Model Construction

**Figure 8:**
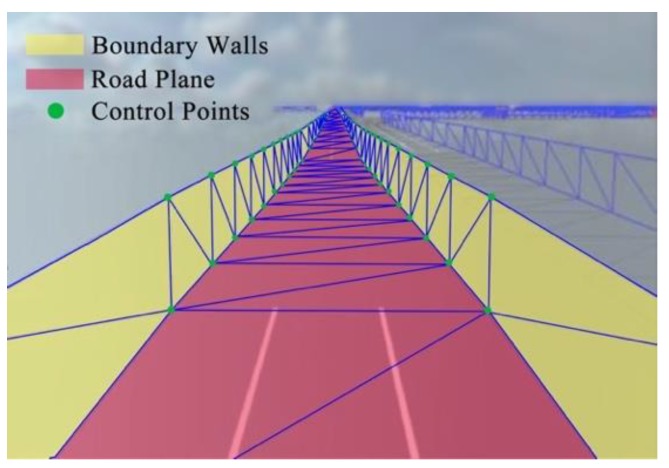
The corridor model and road space.

The 3D road space above the road surface is essential for the motion of traffic elements such as vehicles and pedestrians. Leaving out trees, architectures, nature features, and other traffic elements, the real road surface area can be regarded as a ribbon. Boundary control points define the left boundary wall and the right boundary wall. The road ribbon together with left and right boundary walls makes up a 3D space extends infinitely forward. We define this logical model of road geometric space as “corridor model”, which is shown in Figure 8.

The corridor model is defined as

C = (*L_bottom_*, *L_left_*, *L_right_*), (7)

where *L_bottom_* is the road plane, *L_left_* is the left boundary wall, and *L_right_* is the right boundary wall.

**Figure 9:**
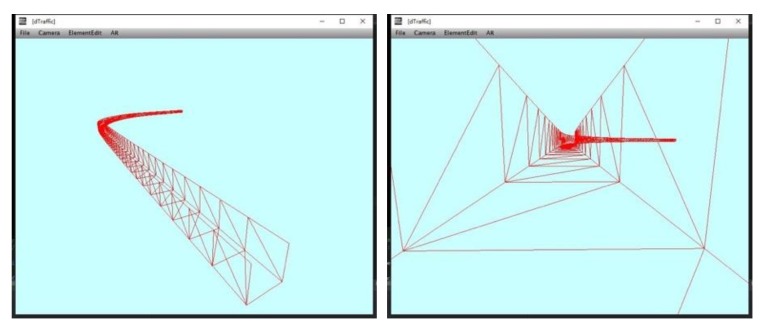
Road geometric space in two different viewpoints.

The road geometric space based on corridor model (shown in Figure 9) can be implemented by the following steps.

- After the road section is assigned, OSMParser analyses the GIS data to obtain sequence of road center points, lane width, and lane number.
- Sequences of the left boundary and right boundary (LeftVertices[] and RightVertices[]) are figured out through calRoadArea using the data obtained from last step.
- Index of triangle meshes Indices[] is set based on the rendering rules of triangle mesh in computer graphics. Indices[i] records coordinates of three vertexes.
- Sequences of boundary points are connected to adjoint triangle meshes which form the road surface.
- Moving road boundary points through the Y axis, we obtain other two sequences of boundary points. The boundary walls in corridor model can be rendered using these two sequences.

### 5.2. Boundary Restraint

**Figure 10:**
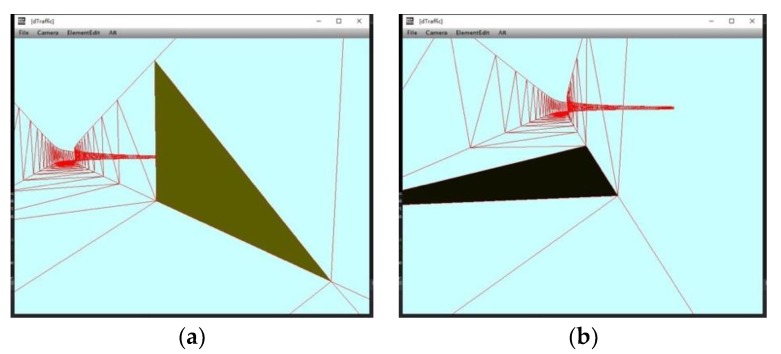
Road collision and boundary wall collision. (**a**) The dark green area shows the collision with boundary wall; (**b**) the black area shows the collision with road surface.

The 3D road geometric space is used to restrict the motion range of traffic elements to be in the operating area. Irrlicht engine provides three methods of collision detection respectively based on ellipsoidal bounding box, triangle, and octree. We choose triangle collision detection to achieve our goal. The scene manager builds a triangle picker to judge whether the ray intersects with the plane of triangle mesh. Then, the triangle picker binds to the scene node waiting for collision detection. For example, when the vehicle moving in the road geometric space collides with the boundary, collision response animator controls the vehicle to response to the collision, so that the vehicle keeps moving on the road surface. Figure 10 shows the results of collision detection of the boundary wall and road.

### 5.3. Registration and Augmentation of Road Scene Data

The perception data of road environment is the data captured by the vehicle sensors, including road scene videos, location information captured by GPS, and pose information from inertial navigation, etc. When existed road scene videos are used for augmentation, no new real-time data is available from the road scene. Thus, we transform the coordinate system of existed perception data to the coordinate system of virtual road space to avoid frequent coordinate transformation between the real world and virtual world. The virtual world uses ENU coordinate system. The transformation relationship can be expressed as

*C_ENU_* = ***RST****C_e_*, (8)

where *C_e_* is the coordinates in real world, *C_ENU_* is the coordinates in ENU coordinate system, ***R*** is the rotation matrix, ***S*** is the scaling matrix, and ***T*** is the translation matrix.

**Figure 11:**
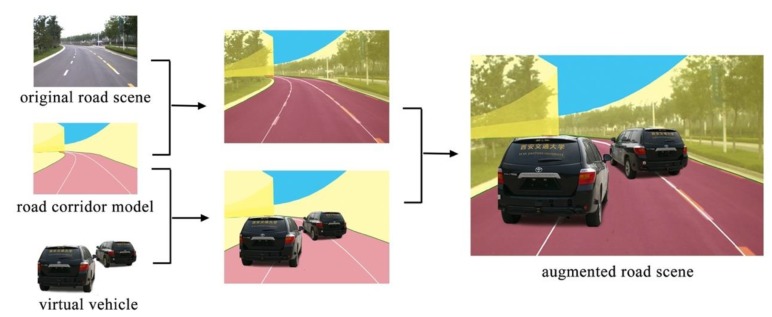
Road scene augmentation based on the corridor model.

After the transformation and registration, virtual camera is one-to-one correspondence to real camera. In the virtual road scene, virtual camera moves through the path of the real camera. Each recorded viewpoint has corresponding road scene image. Then, the virtual vehicle is synthesized to the road scene image, shown in Figure 11.

## 6. Experiments

### 6.1. Dataset of Vehicle Image

**Figure 12:**
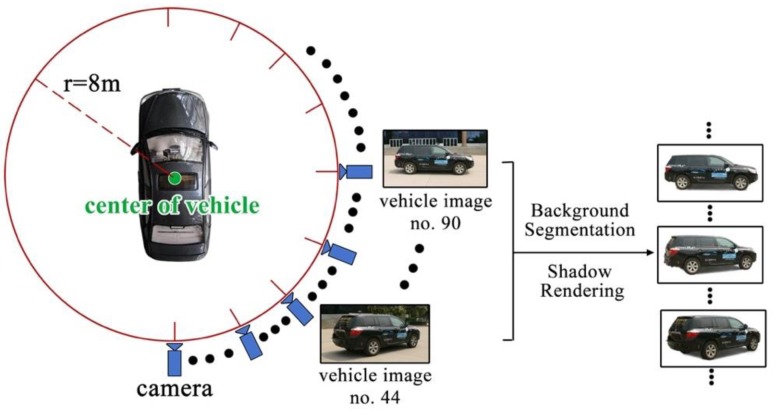
Collection solution of real vehicle images.

Since the acquisition of vehicle image in all viewpoints is unavailable, we discretely capture the image of unmanned vehicle at different viewpoints, as shown in Figure 12. We chose a large square plane without occlusion. The camera moved through a circle centered at the center point of vehicle. The distance between the camera and center of vehicle is 8 m. The height of camera above the ground is 1.8 m, which is the same as the height of camera capturing real road scene videos. We captured one image every other 1°, and obtained a set of 359 images in all. Each image is 2048 × 1536 pixels. Subsequently, we removed the background and rendered shadows manually to obtain a new set of vehicle images in different viewpoints. The new set of vehicle images is indexed by *γ*_1_ and *γ*_2_. The index is organized in the form of hash table to achieve quick load of vehicle images.

**Figure 13:**
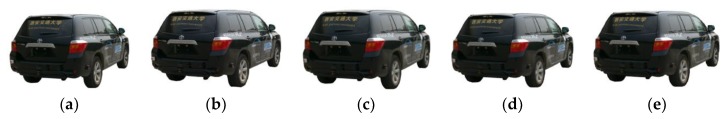
Vehicle image interpolation. (**a**) Image captured at 22°; (**b**) image captured at 24°; (**c**) image interpolated at 22.5°; (**d**) image interpolated at 23°; (**e**) image interpolated at 23.5°.

However, the images we captured are not enough. When the virtual vehicle moves to different angles, the vehicle image switches abruptly. To solve this problem, we need more vehicle images in other viewpoints. For the vehicle images that have not been captured, we generate them by view interpolation [23] and the result is shown in Figure 13.

### 6.2. Miniature Controllable Environment

We design a miniature scene to verify the reasonability and accuracy of our approach. This is because the advantages of using miniature scene are more controllable than large scene. Besides, most of the current research does not deal with comparisons between real-world images and synthesized scenes. The miniature scene can provide us quantitative data to evaluate the approaches. The coordinates, orientation, and size computed by real location data would be compared with those that are obtained directly from the real image. If these two types of data can match well, then the approach is effective.

**Figure 14:**
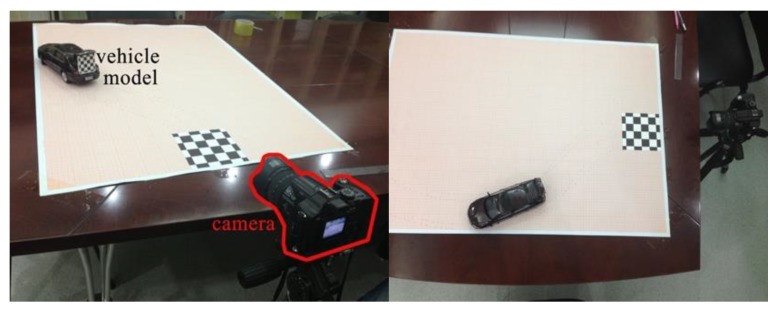
Miniacture controllable experiment environment.

As shown in Figure 14, a piece of graph paper in the size of A0 and printed with a chessboard pattern for calibration was laid on a plane to record real locations. The mesh area in the graph paper is 75 cm × 105 cm. Then, the camera was calibrated. It should be noted that the auto-focusing function of the camera should be shut down to keep the intrinsic parameters of camera in constant. The camera was kept still while a car model with model-to-real scale of 1:18 moving step by step. So, the motion area of the miniature environment is equal to the real road area of 13.5 m × 18.9 m. The chessboard on the back of car model is used to obtain the orientation of car model from the original image.

**Figure 15:**
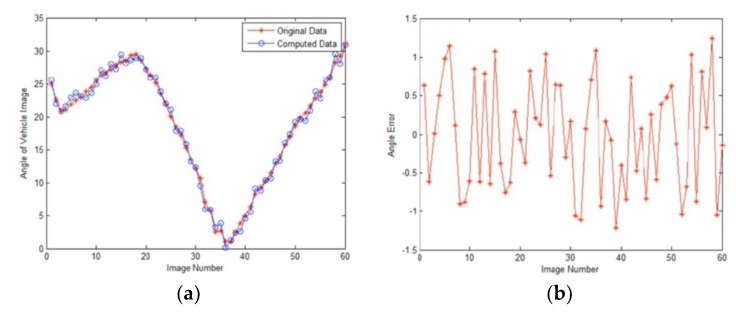
Calculation results of angle. (**a**) Comparison result. The blue line is original data and the red line is our result. (**b**) The error of angle is less than 1.2° and the average error is 0.6°.

**Figure 16:**
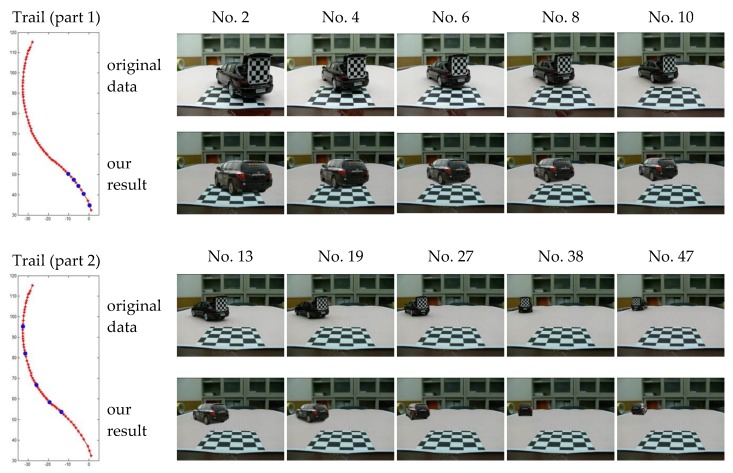
Comparison results of our simulation approach and original image data. The first column shows the trail of vehicle model. The first part of the tail marked with blue dots represent images numbered 2, 4, 6, 8, and 10 from the image sequence, while the second part represent images numbered 13, 19, 27, 38, and 47. In each group of images, the first row shows the original image and the second row shows our simulation results.

An image, the new location coordinate and orientation of the car model were recorded after the car model had been moved to a new place. Thus, we collect 60 groups of images and location data. The location data is used by our approach to compute relevant parameters. Figure 15 and Figure 16 show the comparison results of our algorithm and original data.

### 6.3. Dynamic Simulation in Scene Browser

For the purpose of further verification of our method, we embed the algorithm in a scene browser and choose a typical traffic scene including the intersections for virtual vehicle to execute the driving tasks of lane change, overtaking, slowing down and stop, right turn, and U-turn.

Our experiments were undertaken on a computer with an Intel i5 processor @3.33 GHz and with 16 GB Memory). The experimental data was mostly taken from the TSD-max dataset [24], which was constructed by the Institute of Artificial Intelligence and Robotics at Xi’an Jiaotong University in China. The dataset is composed of road images captured from urban roads, rural roads, highways, etc.

**Figure 17:**
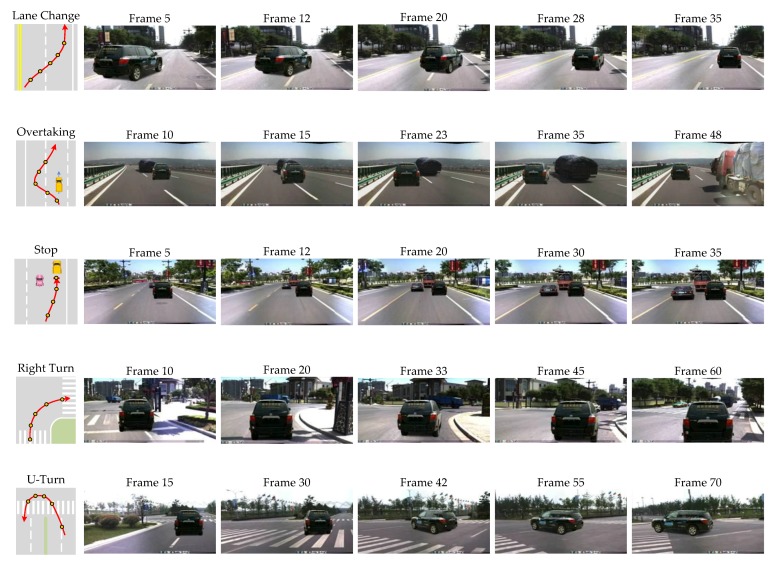
Simulation of typical driving behaviors. The first column shows the driving routes and sampled points of each driving task. Images from the second to sixth columns show the simulation results of sampled points marked in the first column.

The traffic image sequences are formed by frames of videos captured from real road environment. The fame rate of the video is 24 fps. When we collect the videos of real road environment, we get the GPS data at the same time. However, GPS data only record the ground and lacks the height information. The height of viewpoint is manually measured. In our experiment, the height of viewpoint is 1.8 m. The height of virtual vehicle’s center related to the type of vehicle. Thus, GPS data and height data provide the coordinates of virtual vehicle’s center and that of viewpoint corresponding to each image. Applying the algorithm and method mentioned in Section 4 and Section 5, we synthesized and augmented the real road scene video, as shown in Figure 17. The computing time is less than 2 ms. The rendering time is about 17 ms. The all process time is about 28 ms. Since the video frame rate is 24 fps, the all process time can meet the real-time requirement. The motion of augmented vehicle is smooth and realistic.

## 7. Conclusions and Future Work

In this paper, we propose a simple and effective approach of simulating dynamic driving behaviors in the traffic scene organized by image sequences collected from real road environment. In order to obtain the geometric consistency of 3D vehicle model and road environment, we use GPS data to accomplish the registration, obtaining vehicle pose and scale transformation. A logical model named as “corridor model” is defined to restrict the motion range of virtual vehicles. The experimental results verify good performance of our method on simulation of dynamic driving behaviors in typical traffic scenes.

For further work, we will build a more complete simulation system with the function of editing traffic scene freely and easy to use. For example, light and weather condition impacts the performance of visual task for unmanned vehicles. Some detectors based on machine learning, such as CNN-based detectors, highly rely on data augmentation techniques to stimulate performance; training detectors with both day and night images are necessary so as to make them more general. In future, we will generate image data in different light and weather condition via generative adversarial networks for varied scenes.

## Figures and Tables

**Table 1:** Comparison of test methods based on three kinds of data types.

	Field Test	3D CG Simulation	Real Multi-Sensor Data
Time and labor cost	High	Low	Low
Security	Low	High	High
Repeatable test scene	No	Yes	Yes
Repeatable test result	No	Yes	Yes
Environment reality	Real	Simulation	Real
Modifiable environment	Hard	Easy	Easy
Typical Scene